# Characterizing Human Oxidative, Anabolic and Glycolytic Metabolism in Athletes with Extreme Physiologies

**DOI:** 10.1186/s40798-026-00989-z

**Published:** 2026-03-09

**Authors:** Daniela Schranner, Henning Wackerhage, Patrick Weinisch, Jürgen Schlegel, Stephanie Bremer, Johannes Scherr, Werner Römisch-Margl, Annett Riermeier, Otto Zelger, Fabian Stöcker, Anna Artati, Michael Witting, Jan Krumsiek, Martin Halle, Martin Schönfelder, Gabi Kastenmüller

**Affiliations:** 1 Institute of Computational Biology, Helmholtz Zentrum München - German Research Center for Environmental Health, Neuherberg, Germany; 2 Professorship of Exercise Biology, TUM School of Medicine and Health, Technical University of Munich, Munich, Germany; 3 University Center for Prevention and Sports Medicine, University Hospital Balgrist, Zurich, Switzerland; 4 Department of Occupational Health, TUM University Hospital Rechts der Isar, Technical University of Munich, Munich, Germany; 5 Prevention Center, TUM School of Medicine and Health, Technical University of Munich, Munich, Germany; 6 Metabolomics and Proteomics Core, Helmholtz Zentrum München - German Research Center for Environmental Health, Neuherberg, Germany; 7 Chair of Analytical Food Chemistry, TUM School of Life Sciences, Technical University of Munich, Freising-Weihenstephan, Germany; 8 Institute for Computational Biomedicine, Englander Institute for Precision Medicine, Department of Physiology and Biophysics, Weill Cornell Medicine, New York, NY USA; 9 Department for Preventive Sports Medicine and Sports Cardiology, TUM School of Medicine and Health, TUM University Hospital Rechts der Isar, Technical University of Munich, Munich, Germany; 10 DZHK (Deutsches Zentrum Für Herz-Kreislauf-Forschung), Partner Site Munich, Munich Heart Alliance, Munich, Germany

## Abstract

**Background:**

Regular physical activity is known to benefit health but the long-term effects of specific exercise training on human metabolism remain incompletely described. In this study, we comprehensively characterized the blood metabolomes of male athletes with distinct exercise-adapted metabolic profiles, comparing endurance athletes (n = 11), sprinters (n = 8), and natural body builders (n = 9) as models for highly oxidative, glycolytic, and anabolic metabolism, respectively.

**Methods:**

Serum samples of these athletes and a control group of male untrained individuals (n = 7) were collected both at rest and after maximum exercise. Using untargeted metabolomics profiling and weighted correlation network analysis, we examined associations of metabolites and metabolite modules with athlete groups and their characteristic traits (e.g., cardiovascular fitness or muscularity).

**Results:**

Our analyses revealed distinct metabolic signatures for the different groups: a highly anabolic metabolism was characterized by lower levels of sulfated steroids; a highly oxidative metabolism by higher levels of phospholipids; and a highly glycolytic metabolism by lower levels of sphingomyelins. In response to maximum exercise, 130 metabolites changed across all groups (e.g., N-lactoyl amino acids, acylcholines, energy metabolites), while 57 metabolites showed differences in magnitude or direction of change between groups (e.g., fatty acid oxidative products, cortisol).

**Conclusion:**

Our findings demonstrate that exercise-induced adaptations in metabolism distinctly shape the human serum metabolome and influence the metabolic response to exercise. These insights are relevant for diseases driven by dysfunctional metabolism, such as impaired fat oxidation and dysregulated glycolysis (e.g., diabetes, dementia) and muscle wasting (e.g., sarcopenia), where our specialized populations may serve as useful models.

**Supplementary Information:**

The online version contains supplementary material available at 10.1186/s40798-026-00989-z.

## Key Points

- We deeply characterized and compared the serum metabolome (quantification of ≈850 metabolites) of endurance athletes, natural bodybuilders, sprinters, and untrained controls at rest and in response to maximum exercise.
- Specialized long-term training leads to metabolic adaptations in sulfated steroids (bodybuilders), phospholipids (endurance athletes), or sphingolipids (sprinters).
- Regardless of training history, energy metabolites and N-lactoyl amino acids increase after all-out exercise.
- Fifty-seven metabolites changed differently in response to exercise between the athlete groups, highlighting their different metabolic capabilities under physical stress.

**Supplementary Information:**

The online version contains supplementary material available at 10.1186/s40798-026-00989-z.

## Introduction

Exercise induces both short- and long-term physiological changes that are beneficial to health [1, 2]. Depending on the type of exercise, different adaptations occur, including metabolic (e.g., higher fat oxidation rates), morphological (e.g., more muscle mass) and cardiovascular (e.g., increased stroke volume of the heart) changes. Many studies have extensively investigated the short-term metabolic responses to an acute bout of exercise using a metabolome-wide approach [3]. Additionally, differences in these responses across various exercise modes [4] and protocols [5] in human blood metabolomes have been well described.

In contrast, long-term metabolic adaptations—particularly how they depend on exercise modes (e.g., resistance or endurance exercise)—have been less thoroughly studied, despite their relevance to the health benefits of physical activity reported in observational cohorts [6]. To explore exercise mode-specific long-term effects, highly specialized athletes provide good models [7]. Their metabolism has been shaped by years of specific training, allowing them to express metabolic extremes within the general population [8].

In this study, we conducted an untargeted metabolomics analysis in the sera of highly oxidative endurance athletes, highly anabolic natural bodybuilders, highly glycolytic sprinters and untrained subjects as a control group. Unlike previous studies, our approach spans a wide range of metabolic pathways, incorporating numerous metabolites that are not directly involved in energy metabolism. We examined the metabolomes both at rest and following an acute bout of maximum exercise to capture metabolic differences between groups that may only emerge under physical stress. With this analysis, we aimed to answer the following questions: (1) How do highly oxidative (endurance athletes), highly anabolic (natural bodybuilders) and highly glycolytic (sprinters) athletes, who have been training for years in their sports, differ in their blood metabolomes? (2) How do these athlete groups differ in their metabolic response to the same acute exercise?

## Methods

### Ethical Approval

All participants gave their written informed consent before entering the study, which was approved by the Ethics Committee of the Faculty of Medicine of the Technical University of Munich (study identifier: 356/17S).

### Study Cohort and Testing

The cohort comprised 35 healthy male subjects (11 endurance athletes, 9 natural bodybuilders, 8 sprinters and 7 untrained control subjects). Sprinters were recruited from a training group sharing the same training regimens, seasonal competitions, and having similar nutrition. Recruited bodybuilders were members of the same bodybuilder foundation and therefore followed the same rules for the use of dietary supplements and had similar competitional schedule. This recruitment strategy was chosen to facilitate standardization and support compliance of participants with study requests. Due to constraints in budget, time for recruitment, and lack of access to similar female training groups, we restricted our study to male participants.

Study participants underwent cardiopulmonary exercise testing (CPET) to exhaustion on a stationary bicycle ergometer. CPETs were performed to obtain the subjects’ cardiorespiratory fitness (VO_2_max) and to apply a maximum physiological exercise stress as a trigger for metabolic responses. Tests were performed at 8 am in the morning after a 10-h overnight fast. The CPET testing included a three‐minute warm up, followed by a ramp‐test on a bicycle ergometer (Lode, Groningen, Netherlands) with power increasing linearly at a rate of 30 W per minute until voluntary exhaustion. During cycling, we continuously measured gas exchange with a stationary cardiopulmonary exercise testing system (Cortex, Germany) to obtain VO_2_max values. Venous blood samples for metabolomics analysis were taken 10 min before and 5 min after the CPET from an antecubital vein.

After the CPET, participants rested for 90 min and ingested drinks and foods ad libitum. After a re-warm for ~ 15 min (10 min ergometry at 100 W and 5 min supervised jumping and dynamic stretching exercises), we recorded reactive strength (measured by a drop jump from 30 cm height with a force plate (Kistler GmbH, Germany)) and maximum hand grip force (Jamar, JLW instruments, USA). For both measures, the best out of three attempts was recorded, respectively.

Diet and dietary supplement intake were standardized for 24 and 48 h before the tests, respectively, to minimize acute dietary confounders on the metabolome. To ensure that participants met the training volumes of our inclusion criteria (endurance: 10 h/week, bodybuilding: 6 h/week, sprint: 8 h/week, controls < 2 h/week) the participants were asked to report their exercise training for four weeks before the study. They also had to log their diet, dietary supplements and medication intake for one, two and four weeks before the study, respectively, to allow tracing metabolic differences between groups back to potential differences in diet or dietary supplementation.

In total, we collected 22 health- and/or exercise-related traits, including age, weight and body mass index (BMI), measures of muscularity (circumference of the M. biceps brachii and the M. quadriceps femoris), grip strength, reactive strength and relative maximum isometric strength of the M. quadriceps femoris, measures of cardiovascular fitness (e.g., maximum oxygen uptake capacity (VO_2_max), ventilatory threshold 1 (VT1), lactate clearance and relative maximum workload in the CPET (Supplementary Table S1). More details on the study design and the testing procedures can be found in [8].

### Sample Collection and Untargeted Metabolomic Profiling

Venous serum samples were clotted upright for 30 min at room temperature and then centrifuged for 10 min at 15 °C and at 2460*g*. Supernatant sera were harvested, aliquoted into 400 µL cryotubes and immediately put on dry ice. Until analysis, samples were stored at − 80 °C. All samples were measured at the Metabolomics Core Facility at Helmholtz Munich using analytical methods established and validated by Metabolon Inc. (Durham, NC, USA).

On the day of extraction, samples were thawed on ice and 100 µL of serum were pipetted into a 2 mL 96-well plate. In addition to samples from this study, a human reference plasma sample (Seralab, West Sussex, UK) and another reference of human serum (Seralab, West Sussex, UK) were placed in one and four wells of each 96-well plate, respectively. These samples served as technical replicates to assess process variability. Besides those samples, 100 μL of water was placed in four wells of the 96-well plate to serve as process blanks. The 70 experimental samples were measured in two batches, where each batch included 35 experimental samples; thereby samples from the same participant were placed on the same plate (except for one participant); and samples from each athlete group and the untrained control group were distributed across the two batches.

Next, proteins were precipitated and the metabolites in the serum samples were extracted with 500 µL methanol, containing 4 recovery standards to monitor the extraction efficiency. After centrifugation, the supernatant was split into aliquots of 50 µL to be analyzed by three different mass spectrometry (MS)-based methods. These sample extracts were dried on a TurboVap 96 (Zymark, Sotax, Lörrach, Germany). To minimize human error, liquid handling was performed on an automated MicroLab STAR® robot (Hamilton Bonaduz AG, Bonaduz, Switzerland).

All analytical methods utilized a Waters ACQUITY ultra-performance liquid chromatography (UPLC) and a Thermo Scientific Q-Exactive high resolution/accurate mass spectrometer interfaced with a heated electrospray ionization (HESI-II) source and Orbitrap mass analyzer operated at 35,000 mass resolution. Prior to the UPLC-MS/MS runs, the dried extracts were reconstituted with 40 µL of solvents compatible to each of the three methods. Each reconstitution solvent contained a series of standards at fixed concentrations to ensure injection and chromatographic consistency. One aliquot was analyzed using a reverse phase (RP)/UPLC-MS/MS method under acidic positive ion conditions, chromatographically optimized for more hydrophilic compounds. In this method, the extracts were gradient eluted from a C18 column (Waters UPLC BEH C18-2.1 × 100 mm, 1.7 µm) using water and methanol, containing 0.05% perfluoropentanoic acid (PFPA) and 0.1% formic acid (FA). Another aliquot was analyzed using an RP/UPLC-MS/MS method under acidic positive ion conditions, chromatographically optimized for more hydrophobic compounds. In this method, the extracts were gradient eluted from the same aforementioned C18 column using methanol, acetonitrile, water, 0.05% PFPA and 0.01% FA and was operated at an overall higher organic content. Another aliquot was analyzed using an RP/UPLC-MS/MS method under basic negative ion conditions with a separate dedicated C18 column. The basic extracts were gradient eluted from the column using methanol and water, however with 6.5 mM ammonium bicarbonate at pH 8. The MS analysis alternated between MS and data-dependent MS^n^ scans using dynamic exclusion. The scan range varied slightly between methods but covered 70–1000 m/z.

The spectral data, which were recorded at Helmholtz Munich, were sent to Metabolon for further processing, including peak identification, alignment and integration and raw data quality control using their in-house hardware and software (Metabolon, Inc., Durham, USA) [9]. Compounds were identified by comparison to Metabolon’s in-house spectral library, which contains entries of purified standards or recurrent unknown entities. Three criteria were used for matching: retention index (RI) within a narrow RI window of the proposed identification, accurate mass match to the library, and the MS/MS forward and reverse scores between the experimental data and authentic standards (Level 1 identification according to Metabolomics Standardization Initiative (MSI) [10]; n = 677) or repeatedly recorded spectra (Levels 2–4; thereof n = 189 with chemical annotation according to plausibility and comparison with public spectral libraries).

In total, relative quantification (ion counts) were provided for 1020 metabolites of known and unknown chemical identity. Known metabolites cover a broad spectrum of human metabolic pathways, including metabolites from eight major classes (“superpathways”): lipids, amino acids, xenobiotics, peptides, nucleotides, cofactors and vitamins, carbohydrates and energy metabolites. Additional information for each metabolite, such as annotated specific pathways (“subpathways”) and references to metabolite databases including HMDB and PubChem, is provided in Supplementary Table S2.

### Statistical Analysis

#### Data quality control and preprocessing

We first tested if the proportion of missing values of any sample in our dataset was greater than the mean missingness over all samples plus 5 standard deviations (SD) to identify outliers regarding missingness. No sample had to be excluded based on this criterion. To adjust for batch effects in the measured ion counts, we normalized the values of each metabolite to its batch median in the four aliquots of the commercially purchased reference serum. We then calculated the coefficient of variation (CV) across the normalized values for the four reference plasma samples to assess the remaining technical variability of measurements. We excluded 69 metabolites with CV > 25%. Finally, 94 metabolites with more than 30% missingness over all samples were excluded, data were log2 transformed and missing data points were imputed using a k-nearest neighbor approach (k = 10) [11]. To avoid that group differences in the subsequent statistical analysis are driven by extreme values in one or a few subjects, we excluded outlier data points defined as data points higher or lower than the median plus or minus three times the interquartile range of each metabolite (= 601 outliers data points, ~ 1% of all data points). If outliers were identified for one subject in at least one time point (baseline or post-exercise), both data points of that metabolite for that subject were excluded. After outlier removal, we re-imputed the dataset using the same k-nearest neighbor approach as before.

The final dataset included data on 857 metabolites in 70 samples: 362 lipids, 191 amino acids, 109 unknown/partially characterized metabolites, 90 xenobiotics, 33 peptides, 30 nucleotides, 26 cofactors and vitamins, 9 carbohydrates and 7 energy metabolites. All steps of the quality control were performed using the Metabolomics Analysis PipeLinE Toolbox (maplet) package version 1.0.0 [12].

#### Weighted Correlation Network Analysis (WGCNA)

To reduce the dimensionality of our dataset, we clustered metabolites that correlated strongly into so-called “modules” using WGCNA [13]. We first identified the modules separately for baseline and post-exercise datasets applying the R-based WGCN analysis pipeline. This analysis yielded six modules per timepoint. For each module, we kept only those metabolites that had been assigned to the same module at baseline and post exercise to define robust consensus modules. We then determined the eigenmetabolites and sums of metabolite z-scores as representative measures of each module and used them to test for associations with health and exercise-related traits. Each step is described in detail below.

(1) Module detection: We applied the WGCN analysis pipeline [13] to construct a weighted correlation network and define modules of correlated metabolites based on the abundances of the 857 metabolites at baseline using the automated one-step network construction option (*unsigned*, *soft-thresholding power* = 6, minimum number of metabolites within one module was set to 30) and hierarchical clustering. Metabolites were assigned to five color-coded modules of correlated metabolites (“green”, “yellow”, “brown”, “turquoise”, “blue”) or to the “grey” module if no sufficient correlation was found. We repeated the module detection using the same parameters based on the abundances of the 857 metabolites post exercise. To define robust consensus modules, we checked the overlap of metabolites for each pair of matching module at baseline and post-exercise and only kept metabolites that were assigned to the same module at both time points (Supplementary Table S3).

(2) Associating modules to traits: First, we calculated two versions of representative measures for each module: (i) eigenmetabolite: we calculated the eigenmetabolite of a module, i.e., we performed a principal component analysis (PCA) based on the baseline levels of all metabolites in each module. We then used the scores of the first principal component (PC1) as a representative value for each subject within the respective module (= eigenmetabolite). (ii) z-score: we z-scored the values of all metabolites (at baseline) within each module and then calculated the mean of these z-scores within each participant. Associations between the module eigenmetabolites and the 22 exercise- and health-relevant traits were determined using linear models. For visualizing the associations between modules and traits in a heatmap with interpretable effect directions, we used the standardized effect estimates from linear models using the module z-scores as independent variable. Association between the modules and the athlete group assignment (i.e. natural bodybuilders, endurance athletes, sprinters, untrained controls) were tested by ANOVA. For the associated modules, we additionally performed Tukey’s post-hoc tests to determine the associations for the specific athlete groups.

The WGCN analysis was performed using the R package WGCNA version 1.70-3 [13]. The calculation of module eigenmetabolites and the linear models for calculating beta estimates were performed using the R packages ropls version 1.18.8 and lmerTest version 3.1-3.

#### Metabolite changes upon exercise

To test which metabolites changed significantly (p < 5.8E−05 over all subjects and p < 0.05 in each group alone) with acute exercise, we performed paired t-tests on the baseline and post-exercise metabolite abundances based on the data of all subjects, and separately for each athlete group.

#### Group-specific exercise response

We performed an ANOVA (log2 fold change ~ group) on the log2 fold changes between baseline and post-exercise for all 857 metabolites to identify those for which athlete groups responded differently (p < 0.05) to exercise. Additionally, pairwise comparisons were performed to test for differences between the specific groups (p < 0.05).

All figures were created using ggplot2 version 3.3.6. Power calculations were performed using the R package pwr version 1.3.0. All analyses and plots were conducted using R version 3.6.0 and R Studio version 1.3.1093.

## Results

In this study, we analyzed 857 metabolites in the blood serum of 11 endurance athletes, 9 natural bodybuilders, 8 sprinters and 7 untrained controls. Samples were collected at fasted rest and immediately after a graded cycling exercise test to exhaustion. For each participant, we also gathered data on 22 health- and exercise-related traits, including VO_2_max, muscle mass, muscle strength, and weekly exercise time. As expected, the athletes differed significantly in traits most relevant to their respective sports (Table 1). For instance, body mass index (BMI) and biceps muscle circumference were significantly higher in natural bodybuilders compared to all other groups. Cardiovascular fitness (VO_2_max and the maximum load achieved on the bike) was significantly higher in endurance athletes compared to the other groups. Additionally, reactive strength was significantly higher in sprinters compared to all other groups. While these traits were closely tied to the specific sport disciplines (endurance, strength, sprint), some traits were similarly pronounced in more than one group. For example, grip strength was high in sprinters and in natural bodybuilders. Individual values underlying Table 1 are given in Supplementary Table S1.

**Table 1:** Selected health-and exercise related traits (22 in total) of the studied cohort

Measured traits	Control	Natural bodybuilding	Endurance	Sprint
	n = 7	n = 9	n = 11	n = 8
	Mean ± SD	Mean ± SD	Mean ± SD	Mean ± SD
Age (years)	27 ± 3	30 ± 5	30 ± 4	**22 ± 3**^**b,c,e**^
BMI (kg/m^2^)	23.6 ± 2.4	**26.4 ± 2.7**^**e,c,s**^	22.6 ± 1.8	22.2 ± 1.6
Heart rate, rest (bpm)	68 ± 9.4	57.9 ± 5.9	51.1 ± 12.7 ^c^	59 ± 6.8
Heart rate variability (ms)	900 ± 132	1088 ± 66	1207 ± 247 ^c^	1056 ± 99
Biceps circumference (cm)	29.4 ± 2.3	**34.9 ± 3.5**^**e,c,s **†^	29 ± 2.2	28.9 ± 1.7
Quadriceps circumference (cm)	52 ± 5	58.9 ± 5.1^c^	54 ± 4.9	56.1 ± 4
VO_2_max (mL/min/kg)	44.1 ± 4.2	41.1 ± 2.9	**63.9 ± 5.2**^**b,c,s** ∆^	50.7 ± 4.9^c,b^
Max. load achieved, relative (W/kg)	3.8 ± 0.4	3.7 ± 0.3	**5.9 ± 0.4**^**b,c,s**^	4.6 ± 0.4
Lactate clearance (%)**	1 ± 15.6	1.9 ± 40.2	− 31.6 ± 13.5 ^b,c^	− 6.9 ± 22.2
Grip strength (kg)	57.9 ± 11	64.6 ± 8.6^e^	54.6 ± 5.2	60.9 ± 6.2
Reactive strength index*	122.6 ± 28.9	128.4 ± 34.1	149.6 ± 32.4	**205.4 ± 36.7**^**b,c,e#**^
Bodyfat (%)	16.4 ± 6.6^e,s^	10.8 ± 3.6	8.9 ± 2.8^c^	7.5 ± 3^c^
Isometric leg strength (Nm)	293.3 ± 39.4	330.5 ± 71.5^e^	260.6 ± 42.5	321.2 ± 53^e^
Endurance training (min/week)	49 ± 58	60 ± 46	**788 ± 294**^**b,c,s**^	184 ± 63
Resistance training (min/week)	9 ± 21^b,s^	**388 ± 208**^**e,c,s**^	90 ± 41^b,s^	238 ± 64^c,e^
Speed training (min/week)	0 ± 0^s^	70 ± 148^s^	48 ± 66^s^	**281 ± 142**^**b,c,e**^

Significant differences (p < 8.3E−03, α = 0.05/6) from post-hoc pairwise testing between groups are superscripted and those unique for one group are additionally bolded

*Measured by the jump height (cm) divided by the ground contact time (s) after a drop from 30 cm height; indicator of muscular reactive strength

**Capillary lactate 10 min after exercise − capillary lactate 2 min after exercise *100

^b^Significantly different from bodybuilders, ^c^ from controls, ^e^ from endurance athletes, ^s^ from sprinters

^∆, †, #^Evidences of extreme physiologies: ^∆^ with a mean VO_2_max of 65.6 mL/kg/min in the age group 20–29y and 61.8 mL/kg/min in the age group 30-39y, the participating endurance athletes fall within the 99th percentile of VO_2_max of the reference populations [14]; ^†^ characteristic of extreme physiology: 7 out of the 9 natural bodybuilders are in the 95th percentile when compared to the reference data of the corresponding age groups in the population [15]; ^#^ no comparable data from reference populations were available for RSI but participating sprinters have been competing in their sport at a national level

SD: standard deviation; BMI: body mass index; VO_2_max: maximum oxygen uptake capacity

The characteristics of these three athlete groups can serve as (extreme) models for the adaptation of human metabolism to high oxidative (endurance athletes), anabolic (natural bodybuilders), or glycolytic (sprinters) demands of each sport, as their most characteristic traits (e.g., VO_2_max for endurance athletes) represent extreme values (e.g., 99th percentile of VO_2_max) within the population (Supplementary Table S1, Table 1). To characterize these metabolic extremes, we first assessed the differences between the athletes’ metabolomes. In the second step, we analyzed the metabolic response to acute, maximum exercise and tested for differences in this response between the groups.

### Athlete Groups Show Characteristic Differences in Phospholipids, Sulfated Steroid Hormones, and Sphingolipids

**Fig. 1:**
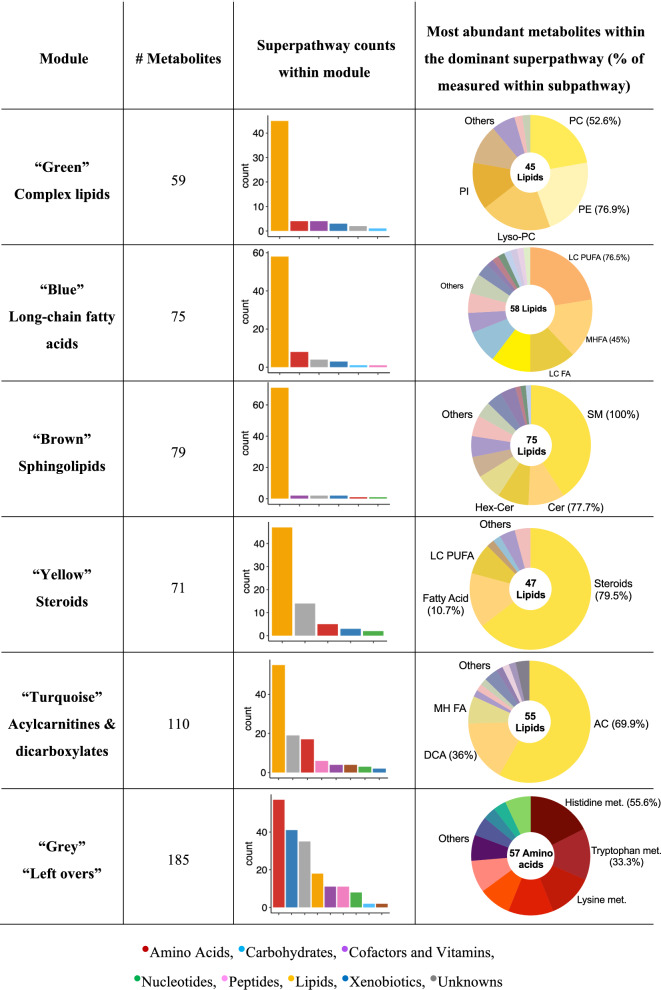
Module characteristics of the six consensus modules that resulted from the overlap between baseline and post-exercise. PC: phosphatidylcholines; PI: phosphatidylinositols; PE: phosphatidylethanolamines; Lyso-PC: lysophosphatidylcholines; LC-PUFA: long-chain poly-unsaturated fatty acids; MHFA: monohydroxy fatty acids; LCFA: long-chain fatty acids; SM: sphingomyelins; Cer: ceramides; Hex-Cer: hexosyl-ceramides; AC: acylcarnitines; DCA: dicarboxylic acids

We characterized the specificities of the groups’ serum metabolomes by performing a weighted correlation network analysis (WGCNA), grouping the 857 measured metabolites, which include 362 lipids, into clusters (“modules”) of highly correlated metabolites. To ensure the robustness of the identified modules, we analyzed baseline and post-exercise data separately and then refined each module to include only those metabolites that were assigned to it at both timepoints (Supplementary Table 3). In total, we identified six modules, each with largely overlapping metabolite sets at baseline and post-exercise (Fig. 1). Each module of highly correlated metabolites is represented by a specific color (e.g., “green”, “yellow”, “brown”), while the “grey” module contains metabolites that showed only weak correlations with other metabolites and, therefore, does not stand for a coherent, biochemically interpretable group. Except for the “grey” module, the identified modules represent different branches of lipid metabolism: complex lipids (e.g., 1-stearoyl-2-oleoyl-GPI (18:0/18:1)), long-chain fatty acids (e.g., docosatrienoate (22:3n6)), sphingolipids (e.g., palmitoyl dihydrosphingomyelin (d18:0/16:0)), steroids (e.g., pregnenetriol disulfate), and acylcarnitines & dicarboxylates (e.g., 2-methylbutyrylcarnitine (C5)).

For testing the associations of modules with athlete groups and exercise or health-relevant traits in an ANOVA, we calculated the eigenmetabolites as representative measures for the “expression” of each module in each participant (for details see Methods). To facilitate interpretation, we also determined the sum of metabolite z-scores for each module and participant for the visualization of effect directions. In total, we found four modules (“yellow”, “green”, “brown”, “grey”) to be associated (p < 0.05) with athlete group assignment (p_yellow_ = 5.9E−07, p_green_ = 0.003, p_brown_ = 0.03, p_grey_ = 0.047), with the associations to the “green” and “yellow” modules being still significant after correction (Bonferroni) for multiple testing (p < 8.3E−03). All six modules showed associations (p < 0.05) with at least one of the 22 exercise or health-relevant traits. Association of endurance-related and bodybuilding-related traits with the “green” and “yellow” modules remained significant after multiple testing correction (FDR), respectively (Supplementary Figure S1, Supplementary Table S4).

**Fig. 2:**
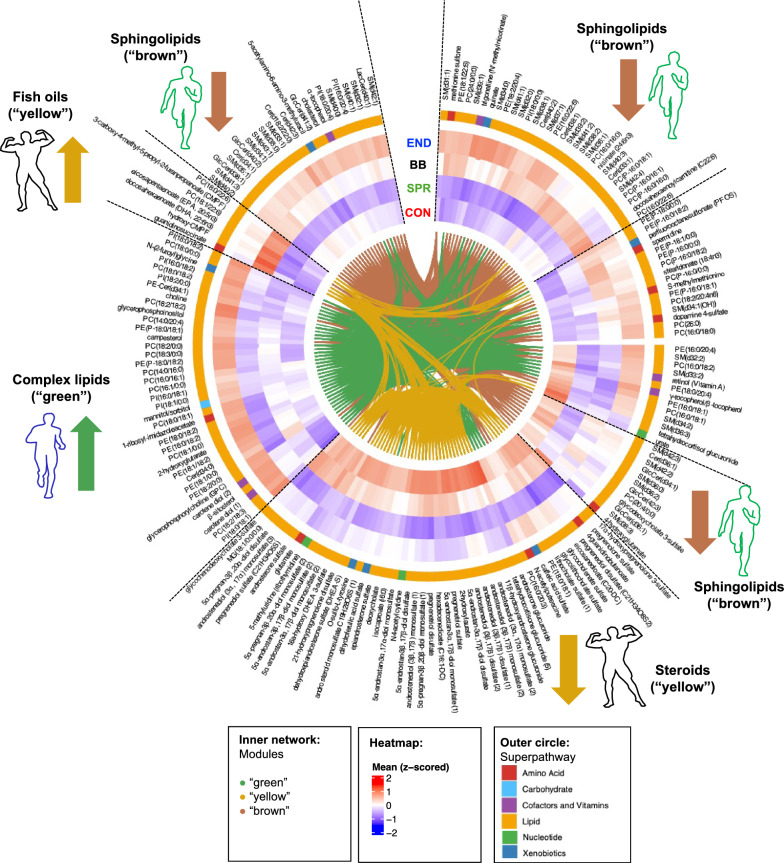
Circular plot showing metabolites within the three modules “green” (complex lipids), “yellow” (steroids) and “brown” (sphingolipids) ( inner network) which are associated with one athlete group each. Baseline metabolite levels per group (as z-scored means) are shown in the heatmap. Superpathways are annotated on the outer colored circle. Unknowns and partially characterized molecules are not shown in this plot. Abbreviations of metabolite names used in this plot are given in Supplementary Table S2

The “green” complex lipids module (see Supplementary Figure S2 and Supplementary Tables S3 and S5 for detailed module characteristics) was significantly associated with traits related to cardiovascular fitness includingVO_2_max (p = 4.3E−04), ventilatory threshold 1 (p = 4.8E−04), relative maximum Watts achieved on the bike (1.6E−04), endurance training time (p = 2.8E−05) (higher). These traits as well as further traits associated at p < 0.05 (lactate clearance (p = 4.0E−03), body fat percentage (p = 0.021) (lower)) are characteristically expressed in well-trained endurance athletes (Table 1). Endurance athletes differed (Tukey’s post-hoc tests) from other groups in the “green” module (p_endurance-bodybuilding_ = 0.004, p_endurance-sprint_ = 0.028, p_endurance-control_ = 0.041). Specifically, endurance athletes showed higher levels of complex lipids, such as phosphatidylcholines (e.g., PE(16:0/18:2), PE(18:0/18:2)) and lysophosphatidylcholines (e.g., PC(18:1/0:0), PE(18:1/0:0)) (Fig. 2, ƒ).

The “yellow” steroids module (for detailed module characteristics see Supplementary Figure S2 and Supplementary Tables S3 and S5) was significantly associated with traits of muscularity, including biceps circumference (p = 3.1E−05), quadriceps circumference (p = 9.6E−04), BMI (p = 1.3E−03) and resistance training time (p = 9.7E−05) (higher), all of which are characteristic for natural bodybuilders (Table 1). Natural bodybuilders differed from the other groups in the “yellow” module (p_bodybuilding-control_ = 7.3E−07, p_bodybuilding-sprint_ = 4.1E−05, p_bodybuildingendurance_ = 0.011). Specifically, natural bodybuilders had lower levels of sulfated steroid metabolites (Fig. 2, Supplementary Table S6) such as androgens (e.g., 5α-androstan-3 α, 17β-diol disulfate) or pregnenolones (e.g., 17α-hydroxypregnenolone 3-sulfate, pregnenolone sulfate) when compared to all other groups. Moreover, docosahexaenoic acid (DHA), eicosapentaenoic acid (EPA), and other metabolites known to be related to fish oil intake (e.g., 3-carboxy-4-methyl-5-propyl-2-furanpropanoate (CMPF), hydroxy-CMPF) were significantly higher in natural bodybuilders.

The “brown” module, which includes all sphingomyelins measured in our study (Fig. 1**, **Supplementary Figure S2, Supplementary Tables S3 and S5), was inversely associated with reactive strength (p = 0.012) and speed training time (p = 0.015), traits that are characteristic of sprinters (Table 1). Sprinters differed significantly from endurance athletes in the “brown” module (p_sprint-endurance_ = 0.024). Sprinters had the lowest levels of sphingomyelins (e.g., SM(d37:1), SM(d39:2)) among all groups (Fig. 2).

As expected, traits specifically expressed in an athlete group were tightly linked to group membership. Consequently, after correcting for athlete group, associations between traits expressed in endurance athletes and the “green” module were no longer significant (p values ranging from 0.057 for ventilatory threshold 1 to 0.43 for endurance training time). Similarly, associations between traits expressed in natural bodybuilders and the “yellow” module (p values ranging from 0.24 for quadriceps circumference to 0.9 for BMI) as well as associations between reactive strength and speed training time (expressed in sprinters) and the “brown” module, were no longer significant (p value for speed training time 0.42 and for reactive strength index (RSI) 0.44) when correcting for the athlete group.

While the “blue” and the “turquoise” modules were not associated with any group, the “blue” module was linked to higher weight (p = 0.028) and the “turquoise” module to lower resistance training time (p = 0.015) and higher systolic blood pressure (p = 0.028).

Finally, the “grey” module was associated with grip strength (p = 0.011) and leg strength (p = 5.82E−03). However, metabolites within this module were not necessarily correlated with each other (i.e., “leftovers” from the other modules). This lack of internal coherence hampers the interpretation of associations at the module level. To capture associations of athlete groups with metabolites in the diverse “grey” module and with metabolites that were not robustly assigned to the same module for pre- and post-exercise measurements, we additionally performed the test for each metabolite separately (Supplementary Table S7). Out of all 857 tested metabolites, 7 and 252 metabolites were significantly associated with group membership after adjusting for multiple testing by controlling the family-wise error rate (Bonferroni correction) and the false discovery rate (FDR; Benjamini–Hochberg correction), respectively. Out of the 7 most strongly associated metabolites, 1,5-anhydroglucitol (p = 8.5E−06), with significantly lower levels in bodybuilders compared to endurance athletes (p = 2.3E−04), was the only metabolite that was not assigned to any of the robust modules.

### In Response to Exercise, Energy Metabolites, N-lactoyl Amino Acids and Hypoxanthine Strongly Increased, Acylcholines and Cysteine Sulfinic Acid Strongly Decreased

**Fig. 3:**
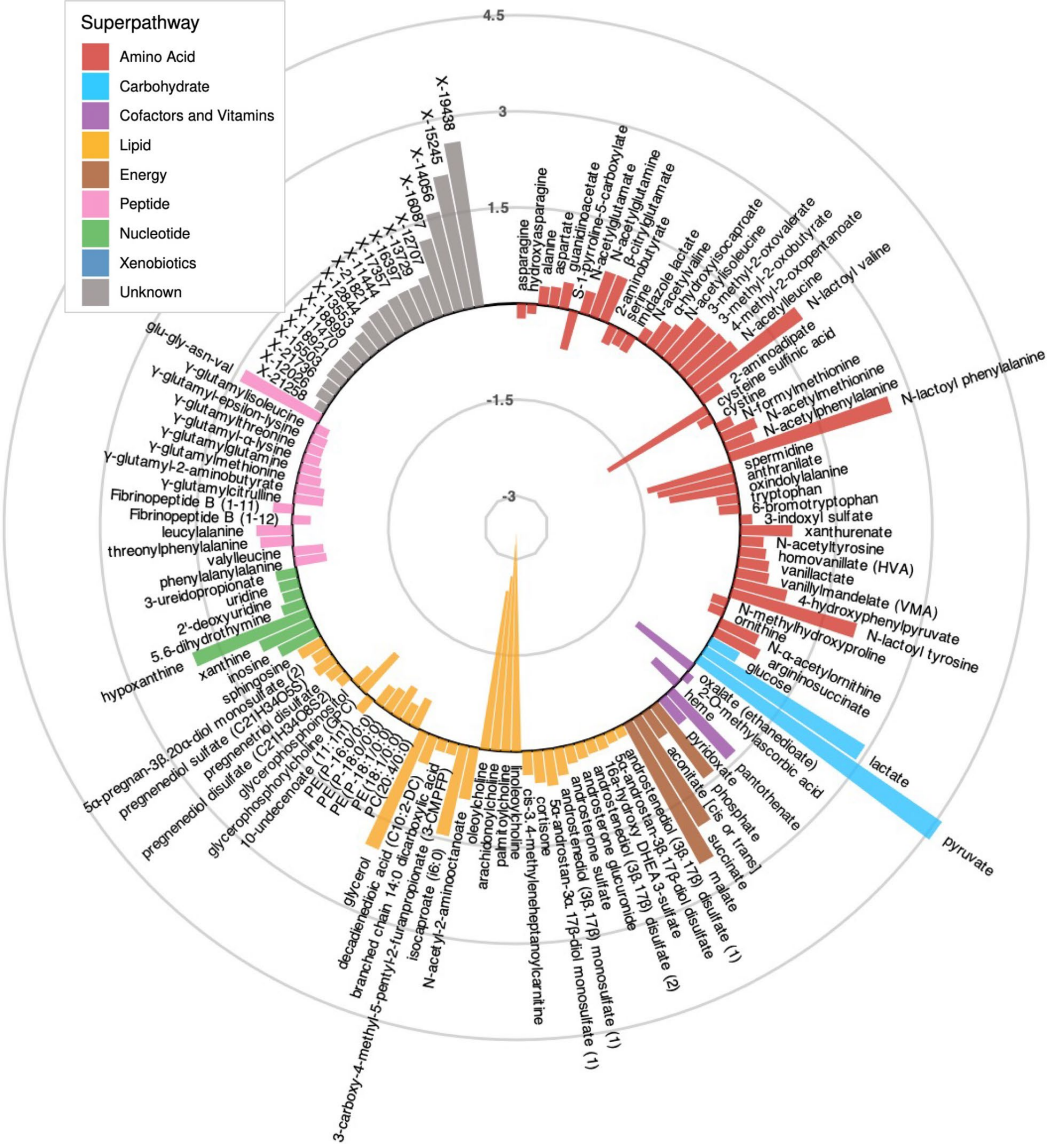
Mean log2 fold changes (over all subjects) of the 130 exercise-responsive metabolites that were consistently changed in each of the four groups. Changes ranged from − 3 to 4.5-log2 fold (log2 fold-change of 1 = doubling of metabolite level in blood; log2 fold-change of − 1 = halving of metabolite level in blood). Metabolite bars are colored by superpathway and sorted by their fold-change within each subpathway

By comparing the baseline and post-exercise metabolite levels in all participants, we found that 225 metabolites were significantly (p < 5.8E−05) changed by exercise after multiple testing correction (Supplementary Table S8). Thereof 130 metabolites were changed with at least p < 0.05 in each group (Fig. 3**, **Supplementary Figure S3).

Metabolites which increased the most were pyruvate (4.6-log2 fold) and lactate (2.9-log2 fold ), followed by three N-lactoyl amino acids, N-lactoyl tyrosine, N-lactoyl phenylalanine, N-lactoyl valine (2.0 to 2.6-log2 fold), energy metabolites, such as succinate and malate (2.0 to 2.5-log2 fold), and glycerol (2.0-log2 fold). We also noticed increases of more than 1.5-log2 fold for hypoxanthine and three so far uncharacterized metabolites, X-19438, X-15245 and X-14056 (Fig. 3).

Metabolites which decreased the most were four long-chain acylcholines (linoleoyl-, palmitoyl-, arachidonoyl-, oleoylcholine; -2.5 to -3.4-log2 fold), and cysteine sulfinic acid (-1.8-log2 fold).

### Metabolite Levels Changed Differently in Response to Maximum Exercise Across Athlete Groups

**Fig. 4:**
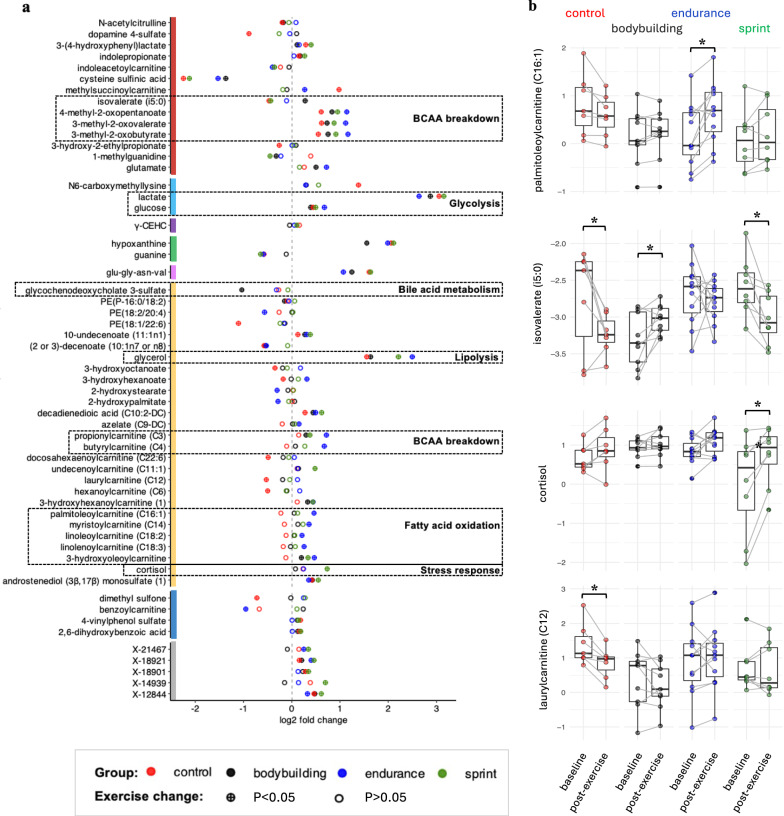
**a** Log2 fold changes of 57 metabolites that showed differential responses (p < 0.05) between groups and changed (p < 0.05) in response to exercise in at least one group. Metabolite classes are color-coded on the y-axis using the same colors as in Fig. 1. Log2 fold changes are colored by subject group and circles filled with a + indicate nominal significance (p < 0.05). Abbreviations of metabolite names are given in Supplementary Table S2. **b** Baseline and post-exercise levels of all participants in the four groups are shown for four selected metabolites: palmitoleoylcarnitine (C16:1) with increases in endurance athletes, isovalerate (i5:0) with increases in natural bodybuilders and decreases in controls and sprinters, cortisol with increases in sprinters, and laurylcarnitine (C12) with decreases in untrained controls

To assess whether the athlete groups differ in their metabolic response to the same acute, maximum exercise, we performed an ANOVA on the log2 fold changes for all metabolites. Sixty-three metabolites showed differential responses at p < 0.05, with the 3 metabolites propionylcarntine (C3), butyrylcarnitine (C4), and palmitoleoylcarnitine (C16:1) reaching multiple testing corrected (FDR) significance level. Out of the 63 metabolites, 57 showed a change (p < 0.05) in at least one of the four groups (Fig. 4a). For example, degradation products of branched-chain amino acids (BCAA) such as 3-methyl-2-oxovalerate, 3-methyl-2-oxobutyrate, 4-methyl-2-oxopentanoate, propionylcarntine (C3), butyrylcarnitine (C4), as well as metabolites linked to fatty acid oxidation (e.g., palmitoleoylcarnitine C16:1 (Fig. 4b), myristoylcarnitine), lipolysis (e.g., glycerol) and glycolysis (e.g., glucose) increased more in endurance athletes compared to the other groups. In contrast, lactate increased less in endurance athletes (Fig. 4a, dotted frames**)**.

Isovalerate, a branched-chain fatty acid, increased in natural bodybuilders but either decreased or remained unchanged in the other groups (Fig. 4b). Similarly, glycochenodeoxycholate 3-sulfate, a bile acid metabolite, decreased in natural bodybuilders but showed no change in the other groups (Fig. 4a). The steroid hormone cortisol which is excreted in response to stress (Fig. 4b), along with an unknown metabolite (X-14939), increased more in sprinters compared to all other groups. Finally, laurylcarnitine and hexanoylcarnitine decreased in controls after exercise, while their levels remained unchanged or increased in the other groups (Fig. 4b).

## Discussion

In this study, we characterized the extremes of human oxidative, anabolic and glycolytic metabolism—three pillars of metabolic health—across three highly specialized athlete groups. We compared the metabolomes of 11 highly oxidative endurance athletes, 9 highly anabolic natural bodybuilders, and 8 highly glycolytic sprinters, all of whom have been training for years in their respective sports and thus, represent extreme models of the corresponding metabolic adaptations and long-term imprint. Additionally, we included an untrained control group for comparison. For our analyses, we used an untargeted metabolomics approach that covers a broad range of metabolic pathways. Significantly expanding previous findings [8], we identified novel, unique characteristics in the metabolomes of these athletes and their response to acute, maximum exercise.

### Highly Oxidative, Anabolic and Glycolytic Athletes Express Unique Metabolomes

In our study, we observed that low levels of sulfated steroids, primarily androsterone and pregnenolone sulfates, were characteristic for highly anabolic natural bodybuilders. In comparison to untrained controls, also the endurance athletes in our study showed lower levels of these metabolites. Steroid hormones are enzymatically sulfated (SULT enzymes) to their inactive (sulfated) forms in the liver, transported to target tissues and de-sulfated again (by STS enzymes) [16]. Different scenarios can be hypothesized to underly an observation of lower blood levels of sulfated steroids: (i) depletion of the blood steroid hormone “pool” (i.e., sum of active and inactive forms) or (ii) lower proportion of sulfated steroids within the blood steroid hormone “pool” (e.g., due to less sulfatation). To differentiate between the two cases, measurements of the active forms of the steroids would be needed but were not part of the metabolomics data generated for our participants. Theoretically, both scenarios would be in line with the assumption of a higher intra-muscular engagement of androgenic steroids (especially testosterone) in (resistance-)trained muscles (e.g., as a consequence of a higher density of androgen receptors [17]) to support muscle growth or maintenance by increasing protein synthesis and activating satellite cells [18]. Various studies have observed acute post-exercise increases of androgenic steroids [19]; but reports on differences in resting concentrations related to resistance training have been inconsistent [17], and the exact role of androgenic hormones in muscle growth is still under debate [20]. How our findings on the inactive forms of androgenic steroids link into the existing knowledge on active steroids cannot be answered based on the available data, warranting further investigation. However, a variant (rs10426201) within the *SULT2A1* gene along with correspondingly lower blood levels of sulfated androgens has recently been linked to elite endurance athletes [21]. While this study did not include bodybuilders or strength athletes, its findings partially support our results showing that well-trained athletes have lower levels of sulfated steroids in their blood compared to untrained subjects.

Besides the lower levels of steroids, the bodybuilders in our study also showed lower levels of 1,5-anhydroglucitol (1,5-AG) in particular when compared to endurance athletes. 1,5-AG is a known marker of short-term glycemic control with lower levels indicating more frequent glycemic excursion over the past days [22, 23]. Its lower levels under hyperglycemic conditions (glucose levels > 180 mg/dL) are a consequence of the competitive inhibition of its reabsorption in the renal tubules [24]. While we are not aware of any previous reports on a link between higher 1,5-AG levels with bodybuilding or related traits such as high muscle mass, worsening of kidney function through long-term high-protein diet and use of vitamin supplements as typical for bodybuilders has been suggested in literature [25, 26] and could underly our observation. However, further research is needed to consolidate these relationships. In addition to these novel findings, we also found higher levels of DHA, EPA, and CMPF—metabolites related to fish intake or supplementation with oils rich in omega-3 fatty acids—in this group, as has been reported previously [8]. The participants did not take any supplements for 48 h before the tests and their diet was standardized for 24 h. Nonetheless, the significantly higher levels of these metabolites probably mainly reflect their higher intake in the weeks before the study as the participants who reported the use of such supplements (3 bodybuilders) or regular fish intake (1 endurance athlete) had the highest levels of these metabolites in blood among all participants.

We also found that endurance athletes had higher circulating levels of complex lipids (e.g., PC, PE, lyso-PC), particularly those with 16–18 carbons in their fatty acid chains, compared to the other groups. This aligns with our previous findings of elevated circulating levels of lyso-PC with 18 carbon fatty acid chains (PC(18:1/0:0) and PC(18:2/0:0)) in a similar but smaller cohort [8]. Increased levels of 16- or 18-carbon fatty acids have been associated with endurance training status and improved oxidative metabolism, such as higher VO_2_max [27], enhanced skeletal muscle oxidative capacity [28] and faster marathon finishing times [29]. We hypothesize that endurance-trained muscles, specialized in oxidative metabolism, use more complex lipids for energy supply than non-endurance trained muscles. Supporting this assumption, the expression of the *PLA2G2A* gene, which encodes an enzyme that catabolizes PC to form lyso-PC, increases after endurance training but not after resistance training [30].

Finally, we observed that lower levels of sphingolipids were characteristic of highly glycolytic sprinters. Sphingomyelins play a role in activating satellite cells, which are precursors to skeletal muscle cells involved in muscle regeneration, and they provide protection against muscle damage [31]. Due to the increased muscular load and activity in sprinting [32], muscular damage may occur more frequently in sprinters. Considering this as a hypothesis, one could speculate that sprinters may consume or store more sphingomyelin in muscles for regeneration processes, consequently reducing the sphingomyelin levels in circulation. Although speculative and not directly deducible from the kind of association results yielded by the present study, this theoretic scenario would be in line with the recently reported increases of muscular sphingomyelin levels after a 12-week high-intensity interval training [33]. However, dedicated experimental work is needed to reveal the actual role of sphingomyelins in the context of sprint training.

Interestingly, many of the metabolites that were different between the here investigated participant groups with their specific training backgrounds have been found associated with various diseases in previous studies. For example, alterations in the levels of lyso-PC, which were highest in endurance athletes in our study, as well as the activity of various phospholipase A2 (PLA2) isoforms catalyzing generation of lyso-PCs have been found associated with diseases such as type 2 diabetes [34] and Alzheimer’s disease (AD) [35, 36]. Specifically, low blood levels of PC(18:2/0:0) [37] and (PC(18:1/0:0) [38] (both highest in endurance athletes) were linked to an increased risk of developing type 2 diabetes previously. Moreover, low levels of PC(18:2/0:0) predicted greater decline of gait speed, a measure used for the definition of sarcopenia, in older adults in the Baltimore Longitudinal Study of Aging [39]. Also, for several of the same phospholipids that we found higher in endurance athletes, lower levels were reported in AD and its precursor stages, in particular PC and PE carrying arachidonic (20:4) or docosahexaenoic acid (22:6) in combination with C16 or C18 fatty acid residues [40–42]. For example, PE(18:1/22:6) and PE(16:0/22:6), which were significantly higher in endurance athletes compared to at least one other group in our study, were significantly lower in subjects with prevalent and/or incident AD in the cohorts investigated by Huynh et al. [40]; analogously, Whiley et al. reported two related PC (PC(16:0/22:6), PC(18:0/22:6)) to be significantly lower in AD cases in two independent cohorts [42]. In recent longitudinal studies investigating the trajectories of lipids in relation to AD phenotypes, steeper decline in PE(18:1/22:6) and the lysophosphatidylcholine PC(18:1/0:0) were associated with conversion to dementia [43]; PC(18:1/0:0) was additionally shown to decline faster with the decline of grey matter volume and thickness [44]. Based on these observations, one may speculate that endurance exercise might help normalize altered phospholipid metabolism in AD. While first studies indeed showed the potential of endurance training to modulate phospholipid metabolism in individuals at risk for AD [45], more research is needed to unveil the exact role of phospholipids and their interrelation with exercise in neurodegenerative and metabolic diseases. As a second example, sphingomyelins and ceramides, for which low blood levels were generally characteristic for sprinters in our study, were suggested as indicators of sarcopenia with higher levels being associated with lower muscle strength [46], in particular palmitoyl sphingomyelin and sphingolipids with long chain fatty acids with 24 carbons (matching SM(d34:1), SM(d42:1), and Cer(d42:3) in our study). Alterations in sphingomyelin and ceramide metabolism have also been implicated in AD (e.g., SM(d34:1) and SM(d43:1)) [47]. While pharmacological modulation of the sphingomyelin metabolism has been proposed for the prevention of both diseases [47, 48], sprint training could be an interesting non-pharmacological option. However, more studies will be needed to test its effectiveness and feasibility as a modulator, especially in elderly.

### Potentially Health-Beneficial N-lactoyl Amino Acids Increase Regardless of Training History

Exercise increases the metabolic demand for energy. In line with previous reports, many metabolites associated with this higher demand increased during our experiment in all groups [4, 5, 49–51]; this included well-described increases in lactate, pyruvate, glucose (due to increased carbohydrate usage [52]), glycerol, malate, succinate (due to increased fat mobilization and TCA cycle activity [52]), and hypoxanthine, xanthine, inosine (due to increased purine degradation [53]). Additionally, we observed the largest decreases in long-chain acylcholines (e.g., oleoyl-, linoleoyl-, palmitoyl, arachidonoylcholine) across all metabolites, matching previous findings by Morville et al. [4]. Similar to our observations, the authors saw significant decreases of those acylcholines immediately after exercise, with a quick return to pre-exercise levels and more pronounced changes in endurance exercise compared to resistance exercise. Comparing metabolite changes triggered by exercise of vigorous versus moderate intensity, Weber et al. recently showed that the decrease of these acylcholines is intensity-dependent with larger decreases being observed at higher intensity [54]. Acylcholines of unsaturated long-chain fatty acids, such as arachidonic, oleic, and linoleic acid, have been proposed as endogenous modulators of the cholinergic signalling system [55, 56], which is crucial for skeletal muscle contraction via the neurotransmitter acetylcholine. While these studies suggest that antagonistic binding of the long-chain acylcholines to nicotinic acetylcholine receptors and the enzyme butyrylcholinesterase underly their impact on the signalling system at cellular level, more research is needed to fully understand its interplay with decreasing blood levels of these metabolites in exercise metabolism. Interestingly, chronically altered blood levels of these unsaturated long-chain acylcholines have been reported in various diseases including type 2 diabetes [38] and cardiovascular diseases [57, 58]. Moreover, an impact of these metabolites on inflammatory processes has been suggested [56]. In contrast to the large effect of acute exercise, neither the acylcholine levels at rest nor their change in response to exercise differed between the athlete groups and untrained controls in our study. Conversely, a previous study by Vike et al. found lower levels of long-chain acylcholines in blood samples of collegiate football athletes post-season when compared to pre-season levels [59]. However, at the same time, the lower acylcholine levels were also associated with more head acceleration events, potentially confounding the observed effect. More studies are required to reveal and explain potential links between exercise-induced changes in acylcholine blood levels and health.

In addition to energy metabolites, various N-lactoyl amino acids showed a strong increase in response to exercise in our study. For the first time, we demonstrate that three N-lactoyl amino acids (N-lactoyl phenylalanine, N-lactoyl valine and N-lactoyl tyrosine) increase significantly after a single bout of maximum exercise, regardless of an individuals’ training history or fitness status. As has been reported previously, the increase of these metabolites does strongly depend on exercise intensity [54] and the type of exercise, with sprint exercise triggering the strongest response in N-lactoyl phenylalanine [60]. Aligning with these results, we did not observe any group-specific differences in the response of the N-lactoyl amino acids to the same maximum exercise (CPET) in our study. But interestingly, the sprinters showed significantly higher levels of N-lactoyl phenylalanine (pre- and post-exercise) when compared to controls and endurance athletes. N-lactoyl amino acids have recently gained attention for their potential role in metabolic health [61]. Specifically, N-lactoyl phenylalanine has been shown to suppress obesity and feeding in mice when administered chronically [60]. While this study reported that mainly N-lactoyl phenylalanine, N-lactoyl leucine, and N-lactoyl valine responded to exercise in mice and racehorses, we demonstrate that N-lactoyl tyrosine is affected by exercise to a similar extent as N-lactoyl valine in humans, suggesting also N-lactoyl tyrosine as a potential modulator of the exercise-associated health benefits (e.g., on body weight).

### An Oxidatively Trained Metabolism Burns More Fat and Protein During Exercise

Across all groups, acute, maximum exercise leads to a mobilization and utilization of various fuels, reflected by increases in metabolites related to lipolysis and fatty acid oxidation and BCAA breakdown. Notably this increase is most pronounced in endurance athletes (Fig. 4a). Since endurance athletes have a higher VO_2_max, they also achieve higher absolute oxygen uptake (VO_2_) at lower intensities compared to individuals with a lower VO_2_max [62]. This higher energy demand at the same exercise intensity explains a more extensive or accelerated mobilization and oxidation of fats and protein, which is reflected in more pronounced increases of related blood metabolites (e.g., glycerol, BCAA degradation products) in endurance athletes compared to the other groups. Our results thereby largely replicate previous findings. For example, Lewis et al. found that exercise-induced increases of glycerol in blood were significantly greater in (non-athlete) subjects with higher VO_2_max [63]. Conversely, subjects with myocardial ischemia showed lowest glycerol changes in response to endurance exercise. Also, the upregulation of protein oxidation and BCAA catabolism triggered by acute endurance exercise has been shown previously [64, 65]; potential consequences on protein intake recommendations for endurance athletes are still under debate [66].

### Pronounced Decrease in Sulfated Bile Acid in Highly Anabolic Natural Bodybuilders

We observed a decrease in the sulfated bile acid glycochenodeoxycholate 3-sulfate in natural bodybuilders, whereas no changes were seen in other groups. Bile acid sulfatation is catalyzed by the same enzyme involved in the sulfatation of steroids [67], supporting our above hypothesis that differences in the regulation of sulfatation might underlie the lower levels of sulfated steroids observed in bodybuilders. Notably, in human muscle, the corresponding unsulfated (and unconjungated) bile acid chenodeoxycholate increased after acute resistance exercise [68]. In case of high anabolic activity, higher needs of bile acids and steroids in their active form (i.e., unsulfated) by the growing muscle might explain the comparably low levels of sulfated (i.e., inactivated) steroids and bile acids in blood.

### Higher Training-Induced Stress Response in Glycolytic Sprinters

Compared to the other groups, sprinters exhibit a more pronounced increase in cortisol levels following acute maximum exercise. This is likely due to years of intense sprint training which typically involves intense bouts of exercise (~ 30–60 s). Such training induces a heightened cortisol response to exercise per se [69, 70]. Cortisol, in turn, stimulates the release of glucose to meet the glucose demand of the highly glycolytic muscles used by sprinters during exercise. This effect appears to be specific to sprint training—and likely sprinters who have more glycolytic muscle fibres—since as neither years of resistance [69] nor moderate endurance exercise [71] induced the same cortisol response. This finding aligns with current research on glucose management, where type 2 diabetics are often advised to engage in high-intensity exercise (in the evening but not in the morning) for improved glycemic control [72].

### Limitations

While using athletes as models helped characterize oxidative, anabolic and glycolytic metabolism in a metabolome-wide fashion, there are several limitations to focusing on male athletes as study groups. First, restricting our study to male participants limits the generalizability of our results, considering known sex-specific differences in metabolism and response to exercise. In general, the characteristic metabolomes observed in athletes, who have undergone years of endurance, sprint, or strength training, need to be validated for broader application. Specifically, it remains unclear whether these findings can be translated to a more diverse, non-athlete population, differing in age, sex, genetic background, and health status, that engages in resistance, endurance, or sprint training. However, on the positive side, concentrating on homogenous groups of athletes who are experts in their respective discipline alongside rigid standardization (e.g., diet the day before testing and sample timing) provided an exceptionally high level of control that is often lacking in human studies.

Second, the exercise test chosen for this study (CPET) involved a bout of exhaustive endurance exercise. Since endurance athletes are better adapted to this type of activity [1, 2], our results on group-specific metabolic changes might be biased toward pathways that function more efficiently in endurance athletes (e.g., fat oxidation) as compared to other groups. While Contrepois et al. have shown that such standardized maximum exercise is sufficient to reveal phenotypic differences in metabolism [3], other exercises such as sprinting or strength exercise probably would have stimulated other branches of metabolism [4] and may have uncovered further group‐specific changes in the metabolome.

A further limitation of our association analysis between athlete traits and metabolite modules is that the traits are closely tied to the group membership (e.g., VO_2_max is high in endurance athletes). As a result, we cannot distinguish whether the group membership or the trait itself is driving the observed associations (e.g., the green module is associated with VO_2_max and also with the endurance athlete group). Analogously, our finding on higher DHA and CMPF levels in natural bodybuilders suggests that some of the identified metabolic differences between groups might be linked to group-specific dietary habits rather than physiological characteristics despite the high level of diet standardization in our study. While these findings underscore the importance of standardization, they also indicate that controlling diet for 24 h and abstaining from supplements for 48 h before the study might be too short to avoid residual dietary influence on the metabolome. However, to standardize diet for longer periods might be challenging for highly competitive athletes.

Our study also comes with limitations regarding the statistical analysis. First, with data on only 35 subjects the sample size of our study is small, limiting statistical power. According to power calculations, we can only identify large group differences (effects with Cohen’s f > 0.743 in the ANOVA-testing of the WGCNA-based metabolite modules) with sufficient (80%) power. While we expect the effects in our study to be large by design due to the extreme physiologies of the participating athletes, larger sample sizes will be needed to identify medium and small group differences. Second, the small samples size along with the high dimensionality of metabolomics data leads to a high multiple testing burden in univariate analyses and carries the risk of overfitting when using multivariate approaches. Using the described combination of WGCNA and hypothesis testing, where the dimensionality reduction step (i.e., definition of metabolite modules) is separated from the testing of group differences (i.e., ANOVA for metabolite modules) reduces the risk of overfitting compared to approaches such as Orthogonal Partial Least Squares Discriminant Analysis (OPLS-DA), where dimensionality reduction and differential analysis are intertwined and directly use the group information. Nonetheless, to prove the robustness of the here identified associations between metabolite modules and athlete group, replication in an adequate independent cohort is required. Another downside of the chosen WGCNA approach is that not necessarily all metabolic pathways/metabolite classes are equally represented by functionally interpretable modules, with the risk of bias towards specific metabolite classes (as lipids in our study) and the risk to miss group-specific metabolic differences in underrepresented branches of the metabolism, especially if their metabolites are assigned to the inhomogeneous “grey” Leftovers module that does not allow any coherent, pathway-like interpretation.

Finally, while this study shows associations, which may help in the generation of mechanistic hypotheses, it does not establish any mechanistic links. Also, caution is important in the interpretation of effect directions, especially when associations with metabolites show opposite effects in diseases and exercise. For example, higher levels of a metabolite being associated with a disease does not necessarily mean that lowering the levels of this metabolite would be beneficial; the higher levels might indicate a natural balancing mechanism and therapeutically increasing this metabolite might be the better choice. Future studies should investigate metabolite fluxes and aim to test causal relationships between exercise-induced adaptation and metabolite alterations.

## Conclusion

This study provides insights into the specific metabolic adaptations of endurance athletes, sprinters, natural bodybuilders, and untrained individuals, highlighting their unique metabolomic signatures. Using the three athlete groups as proxies for how regular, structured exercise modulates metabolism, we show that enhancements in fat oxidation (endurance athletes), glycolytic metabolism (sprinters), and muscle growth (bodybuilders) are linked to adaptations in phosphatidylcholine, sphingomyelin, and steroid metabolism, respectively. Our findings may be particularly relevant for diseases characterized by metabolic dysregulation in these branches of metabolism, such as diabetes, dementia (impaired glucose & lipid metabolism) or sarcopenia (disrupted muscle growth). As metabolic disruptions contribute to the rising burden of non-communicable diseases in an aging society, designing and testing exercise interventions that target specific metabolic pathways—whether to fine-tune metabolism in the general population as a preventive measure or to restore balance as a therapeutic approach—represents a promising direction for future research.

## Supplementary Information

Supplementary material 1. Supplementary Table S1. Phenotypic traits (health, training and dietary supplement intake) measured in our study groups for each individual. Supplementary Table S2. Characteristics of 1020 quantified metabolites including metabolite missingness and coefficient of variation (cv) before and after batch correction. Supplementary Table S3. Module overview for the resulting six modules (only metabolites that overlap in baseline and post-exercise) including metabolite superpathways, subpathways, module membership and significance (referring to baseline metabolite levels) of the module membership. Supplementary Table S4. Module associations. (A) Results of a linear model to associate metabolite modules to phenotypic traits. Nominally significant associations (directionality and strength shown by beta estimates) are bolded (p < 0.05) and significant associations after correcting for testing 6 modules and 22 traits (FDR) are bolded and in red. (B) Results from a linear model to associate metabolite modules to the group and the subsequent post-hoc pairwise comparison to find out which group drives potential associations. Bold font (p < 0.05) and bold red font (p < 0.05/6) indicate nominal and multiple testing corrected significance levels, respectively. Supplementary Table S5. Network connectivity data for the "green", "yellow" and "brown" module. The connection between metabolites is given in columns "from node" and "to node" and the strength of the connection is given by the weight. This data supplements Fig. 3 (inner circle showing network connectivity) in the manuscript. Supplementary Table S6. Z-scored mean baseline (pre-exercise) metabolite values per group, with respective superpathway and subpathway information used for the circular heatmap in Fig. 2. Supplementary Table S7. Results from ANOVA testing of group differences for each metabolite and subsequent group-wise comparisons (Tukey's Post-Hoc tests). Supplementary Table S8. Metabolite response to exercise and group-specific response differences. Supplementary Figure S1. Heatmap showing the associations between modules and health- and exercise-relevant traits. The directionality of association is given by the coloring (red = positive association, blue = negative association) and the strength of association is given by the beta estimate value (color grading). Supplementary Figure S2. Metabolite superpathways and their respective subpathways and an exemplary network extract from the most abundant metabolite subpathway are given for the "yellow", the "green" and the "brown" module. The network shows metabolites (as nodes) and their correlation strength (Pearson correlation) as edges. Thicker edges indicate higher correlation. *note that only a fraction of phosphatidylcholines, sphingomyelins and steroids is shown. Supplementary Figure S3. Volcano plots showing metabolites significantly changed by exercise within each group. Metabolites with nominally significant changes (p < 0.05) from baseline to post-exercise are colored by their superpathway and metabolites with a fold change > 1 or < − 1 and with a p-value < 5.8E−05 (corrected for multiple testing (Bonferroni)) are additionally labelled.

## Data Availability

The dataset generated and analyzed in the current study can be downloaded from the MetaboLights repository for metabolomics data (https://www.ebi.ac.uk/metabolights/MTBLS12814). The R code used for the main analyses and generation of plots is available at https://github.com/SystemsMetabolomics/MetaExtreme.

## Abbreviations

BCAA: Branched-chain amino acid
CPET: Cardiopulmonary exercise testing
BMI: Body mass index
VO_2_: Oxygen uptake
VO_2_max: Maximum oxygen uptake capacity
VT1: Ventilatory threshold 1
UPLC: Ultra-performance liquid chromatography
ESI: Electrospray ionization
HESI: Heated electrospray ionization source
MS: Mass spectrometry
MS/MS: Tandem mass spectrometry
RP: Reverse phase
PFPA: Perfluoropentanoic acid
RI: Retention index (RI)
SD: Standard deviations (SD)
CV: Coefficient of variation (CV)
WGCNA: Weighted correlation network analysis
PCA: Principal component analysis
PC1: First principal component
ANOVA: Analysis of variance
PC: Phosphatidylcholines
PI: Phosphatidylinositols
PE: Phosphatidylethanolamines
Lyso-PC: Lysophosphatidylcholines
LC-PUFA: Long-chain poly-unsaturated fatty acids
MHFA: Monohydroxy fatty acids
LCFA: Long-chain fatty acids
SM: Sphingomyelins
Cer: Ceramides
Hex-Cer: Hexosyl-ceramides
AC: Acylcarnitines
DCA: Dicarboxylic acid
DHA: Docosahexaenoic acid
EPA: Eicosapentaenoic acid
CMPF: 3-Carboxy-4-methyl-5-propyl-2-furanpropanoate
RSI: Reactive strength index
SULT: Sulfotransferase
STS: Steroid sulfatase
